# Functional phosphatome requirement for protein homeostasis, networked mitochondria, and sarcomere structure in C. elegans muscle

**DOI:** 10.1002/jcsm.12196

**Published:** 2017-05-15

**Authors:** Susann Lehmann, Joseph J. Bass, Thomas F. Barratt, Mohammed Z. Ali, Nathaniel J. Szewczyk

**Affiliations:** ^1^ MRC/Arthritis Research UK Centre for Musculoskeletal Ageing Research, Medical School University of Nottingham, Royal Derby Hospital Derby DE22 3DT UK

**Keywords:** Phosphatase, C. elegans, Sarcomere, Proteostasis, Protein degradation, Muscle

## Abstract

**Background:**

Skeletal muscle is central to locomotion and metabolic homeostasis. The laboratory worm Caenorhabditis elegans has been developed into a genomic model for assessing the genes and signals that regulate muscle development and protein degradation. Past work has identified a receptor tyrosine kinase signalling network that combinatorially controls autophagy, nerve signal to muscle to oppose proteasome‐based degradation, and extracellular matrix‐based signals that control calpain and caspase activation. The last two discoveries were enabled by following up results from a functional genomic screen of known regulators of muscle. Recently, a screen of the kinome requirement for muscle homeostasis identified roughly 40% of kinases as required for C. elegans muscle health; 80 have identified human orthologues and 53 are known to be expressed in skeletal muscle. To complement this kinome screen, here, we screen most of the phosphatases in C. elegans *.*

**Methods:**

RNA interference was used to knockdown phosphatase‐encoding genes. Knockdown was first conducted during development with positive results also knocked down only in fully developed adult muscle. Protein homeostasis, mitochondrial structure, and sarcomere structure were assessed using transgenic reporter proteins. Genes identified as being required to prevent protein degradation were also knocked down in conditions that blocked proteasome or autophagic degradation. Genes identified as being required to prevent autophagic degradation were also assessed for autophagic vesicle accumulation using another transgenic reporter. Lastly, bioinformatics were used to look for overlap between kinases and phosphatases required for muscle homeostasis, and the prediction that one phosphatase was required to prevent mitogen‐activated protein kinase activation was assessed by western blot.

**Results:**

A little over half of all phosphatases are each required to prevent abnormal development or maintenance of muscle. Eighty‐six of these phosphatases have known human orthologues, 57 of which are known to be expressed in human skeletal muscle. Of the phosphatases required to prevent abnormal muscle protein degradation, roughly half are required to prevent increased autophagy.

**Conclusions:**

A significant portion of both the kinome and phosphatome are required for establishing and maintaining C. elegans muscle health. Autophagy appears to be the most commonly triggered form of protein degradation in response to disruption of phosphorylation‐based signalling. The results from these screens provide measurable phenotypes for analysing the combined contribution of kinases and phosphatases in a multi‐cellular organism and suggest new potential regulators of human skeletal muscle for further analysis.

## Introduction

Skeletal muscle is required for locomotion and maintaining posture and gait. These roles are facilitated by the actin/myosin‐based contractile units. Frequently, the clinical focus on loss of muscle function is on the loss of locomotor function, for example, with trauma or age or in the muscular dystrophies. In the USA, the costs associated with such musculoskeletal conditions were estimated at 5.73% of the GDP in 2011, up from 3.43% in 1998, and expected to continue to rise as the population continues to age.^1^ However, the establishment, maintenance, and operation of the contractile units require substantial metabolic input. This explains why a muscle is a major contributor to overall metabolic homeostasis both as the major site of glucose storage and disposal and as the main protein/nitrogen reserve. Disruption of muscle glucose disposal likely contributes to the larger public health crisis of type II diabetes,^2^ and the loss of muscle protein seen in various clinical conditions such as burns, sepsis, and cancer can be the proximal cause of death.^3^ Thus, muscle has multiple functions of important clinical relevance.

**Figure 1:**
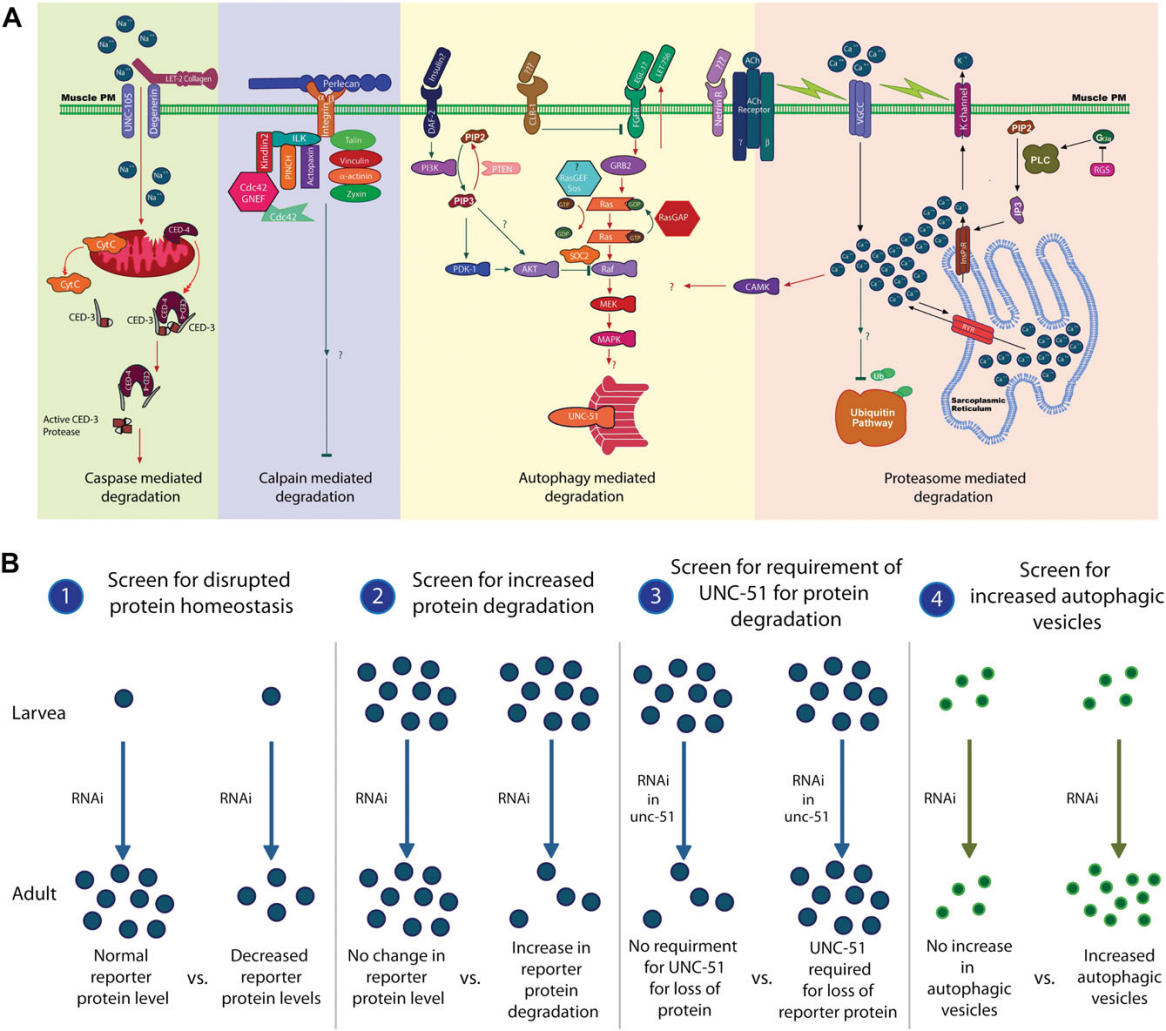
Current model of control of cytosolic muscle protein degradation in Caenorhabditis elegans and schematic of the RNA interference screen for genes potentially regulating autophagy in Caenorhabditis elegans muscle. (A) The model is only from studying the degradation of a single transgenically encoded reporter protein. Far left (green): caspase activation is induced by mitochondrial dysfunction, which can be caused by loss of degenerin channel contact with collagen in the extracellular matrix.^19^ Left (violet): degradation by calpains is regulated by integrin attachment to the basement membrane,^18^ and a significant portion of the integrin adhesome appears to contribute to this regulation.^16^ Middle (yellow): autophagic degradation is controlled by a balance of signal from insulin/insulin‐like receptor (negative regulator, green lines) and autocrine fibroblast growth factor signal (positive regulator, red lines).^12^, ^13^, ^14^ Calcium overload, signalling via CaMKII, also promotes autophagic degradation^17^ as does knockdown of a number of kinases.^20^ Right (pink): intracellular calcium controlled by a combination of membrane depolarization, and G‐protein signalling events are required to negatively regulate proteasome‐based degradation.^15^, ^17^ Displayed model is adapted from models published in Shephard *et al.* 17 and Gaffney *et al.* 19 (B) A schematic of the full RNA interference screen can be found in the kinase screen^20^ which this phosphatase screen is based upon. Briefly, for identification of phosphatase, genes whose knockdown induced autophagic protein degradation was achieved through four steps: (1) genes for which RNA interference produced decreased amounts of reporter protein in muscle were identified. (2) RNA interference against genes identified in (1) was applied to fully developed adult animals to identify RNA interference treatments that produced degradation of the reporter protein in a muscle. (3) RNA interference against genes identified in (2) was applied to fully developed adult *unc‐51* mutant animals to identify RNA interference treatments that failed to produce degradation in the absence of functional UNC‐51. (4) RNA interference against genes identified in (3) was applied to fully developed adult animals containing GFP tagged LGG‐1 to identify RNA interference treatments that produced elevated levels of autophagic vesicles.

Like many clinical problems, the establishment and maintenance of muscle homeostasis are studied not only in human subjects but also in laboratory animals. The worm Caenorhabditis elegans is one such animal. Its small size, transparency, and rapid development coupled with the genetic and genomic tools available make it an ideal model for foundational studies.^4^ The worm has been used to study muscle development,^5^ muscular dystrophy,^6^ fat metabolism,^7^ sarcopenia,^8^ spaceflight‐induced changes in muscle,^9^ and muscle protein degradation (*Figure* 1A);^10^, ^11^, ^12^, ^13^, ^14^, ^15^, ^16^, ^17^, ^18^, ^19^ in each instance, the uncovered genes, signals, and/or underlying concepts of control mechanism(s) have been found to have direct relevance to the same processes and/or conditions in humans.

Three recent kinome‐wide RNAi screens performed in C. elegans to identify the kinome requirement for normal muscle development and homeostasis^20^ identified roughly 40% of the kinome as being important for establishing and/or maintaining proteostasis, mitochondrial structure, or sarcomere structure in muscle. Of these kinases identified in C. elegans, 80 have identified human orthologues and 53 are known to be expressed in skeletal muscle. To complement this data set and to study phosphatases on a genome‐wide scale, we undertook a systematic analysis of phosphatases required for establishing or maintaining muscle cell health in C. elegans. For this study, we employed RNAi to systematically knockdown most individual phosphatases in the C. elegans genome. RNAi was utilized because of both the lack of specificity of available protein phosphatase inhibitors as well as the lack of inhibitors for most of the phosphatome.

## Methods

### Nematode handling and RNA interference screening

Nematode handling, strains utilized, RNAi screening, epistasis testing of identified genes against known protein degradation pathways, and assessment of autophagic vesicles via transgenic reporter protein were all as previously described and diagrammed for the RNAi screen of the C. elegans kinome requirement for a muscle.^20^

A screening list of phosphatase‐encoding genes was constructed from a C. elegans RNAi phosphatase list of 167 genes supplied by Source BioScience LifeSciences Ltd. (Nottingham, UK) and a list of 207 genes supplied by Plowman *et al.*,^21^ the latter of which was based on a genome‐wide HMM search for phosphatase motifs in the C. elegans genome. Comparison of the lists yielded 106 genes that were represented in both lists. The remaining genes unique to one of the two lists were further examined for phosphatase annotation in www.wormbase.org.^22^ Thereupon, a further 67 genes from the Plowman list and a further 25 genes from the Source BioScience LifeSciences Ltd. list were found. Thus, a total of 198 phosphatase‐encoding genes (106 matches +25 Source Bioscience Ltd. +67 Plowman) were collated from both. Where possible, sequence verified RNAi clones against each individual phosphatase were obtained from either of two previously constructed genome‐wide RNAi bacterial feeding clones.^23^, ^24^ These clones were obtained from Source BioScience (Nottingham, UK). After sequence verifying all positive results from our screen, we identified that previously utilized, sequence verified, RNAi constructs were available for 183 putative phosphatase‐encoding genes (see Supporting Information Data S1).

Quality control of our RNAi screens was as previously described and diagrammed for the RNAi screen of the C. elegans kinome requirement for a muscle.^20^ By comparing the developmental phenotypes, such as growth or uncoordinated movement observed in this study to developmental phenotypes observed in RNAi experiments by other investigators using the same RNAi bacteria clone, a potential discrepancy of RNAi results for 17% of total genes screened was identified. This is in concordance with published RNAi screens^17^, ^20^ and half of these potential discrepancies are cases in which we identified a developmental phenotype in response to RNAi but for which a wild‐type phenotype was observed in RNAi experiments by others, indicating that either the RNAi was more effective in this study and therefore these results may be new findings, or these results are false positives. This is again consistent with published RNAi screens^17^, ^20^ and most likely represents our method producing a first discovery of function rate that is higher than past studies. Technical details, including why our false positive rates are lower and first discovery rates are higher than past studies, can be found elsewhere.^25^

### Network analysis

Data from meta‐analyses of physical and functional interactions between the genes identified during the chronic and acute RNAi screen were extracted manually from the following databases: WormBase,^22^ GeneMANIA,^26^ and PhosphoPOINT.^27^ Only interactions between the genes identified to potentially regulate a specific process were searched to construct process‐specific network models. To use PhosphoPOINT data, a human orthologue for the gene identified was searched. The assignment of orthology was taken from a recent meta‐analysis^28^ and review^29^; orthologies used are in Data S1 for phosphatases and in Lehmann *et al.* 20 for kinases. PhosphoPOINT data for the human orthologues were then converted back to the C. elegans orthologues. Some of the genes identified had the same human orthologue and therefore appear as one node in the networks (see Supporting Information Data S2 and S4); these genes are *egl‐4* and *pkg‐2*; *kin‐14* and *frk‐1*. All extracted interactions were visualized using CytoScape.^30^ All extracted data are available for use and similar visualization (see Supporting Information [Link], [Link]); data are divided by individual networks. Data for physical networks are from C. elegans genome‐wide known physical interactions and predicted physical interactions based upon known physical interactions of orthologues in a different species (human, rodent, fly, yeast) both which were retrieved from WormBase and GeneMANIA, as well as on kinome‐wide biochemical data for directly interacting human orthologues, which were retrieved from PhosphoPOINT. Data for functional networks are mainly based on kinome‐wide biochemical data of shared substrates and/or interacting phosphoproteins for the human orthologue derived from PhosphoPOINT. These networks also contain C. elegans known gene product interactions and predicted gene product based upon known gene product interactions for the orthologue in a different species, both which were retrieved from WormBase and GeneMANIA.

### Western blot

For western blot analysis of MEK phosphorylation, 30 worms were picked into 20 μl sterile ddH_2_O and immediately frozen in liquid nitrogen and stored at −20°C. Later the same week, 8 μL of 3× Laemmli buffer was added to each sample and heated for 5 min at 95°C in a hot block, whereupon they were vortexed for 30 s and centrifuged for 1 min and placed on ice. The entirety of each sample was then loaded into a 12% Bis‐Tris SDS PAGE gel (Bio‐Rad, Hemel Hempstead, UK) for electrophoresis for 1 h at 200 V. Separated proteins were transferred onto a PVDF membrane (Bio‐Rad) for 45 min at 100 v, then placed in 3% bovine serum albumin (BSA) in Tris‐buffered saline and 0.1% Tween‐20 (TBST) for 1 h at room temperature. Membranes were washed 3× for 5 min in TBST then incubated at 4°C overnight in primary antibody solution. Anti‐P‐MEK 1/2^Ser 217/221^ (no. 9121) (Cell Signalling Technology, Beverly, MA, USA) was diluted 1:1000 in TBST. Afterwards, the membrane was washed 3× for 5 min in TBST before incubation in the secondary antibody solution of 3% BSA in TBST containing HRP conjugated anti‐rabbit secondary antibody (Cell Signalling Technology), 1:2000 for 1 h at room temperature. The membrane was then washed 3× in TBST, before incubation for 5 min in enhanced chemiluminescence reagent (Millipore, Watford, UK) and visualized using a Chemidoc XRS system. Band volumes were quantified using ImageJ (NIH).

## Results

### Phosphatases required for establishing or maintaining muscle health

To establish the role of each phosphatase‐encoding gene in the genome of C. elegans in establishing and/or maintaining muscle homeostasis, we obtained a set of RNAi constructs against phosphatases from Source BioScience LifeSciences Ltd. and also RNAi constructs against phosphatases identified using a hidden Markov model (HMM) search for phosphatase motifs in the C. elegans genome.^21^ This lead us to identify 198 putative phosphatase‐encoding genes of which 106 were identified by both sources, 25 were unique to Source Bioscience, and 67 were unique to the HMM search; sequence verified RNAi constructs were available for 183 of these genes. Utilizing these 183 RNAi constructs, we repeated the RNAi screening protocol used to identify kinases required for normal muscle proteostasis, protein degradation, mitochondrial structure, and sarcomere structure (diagrammed in Lehmann et al.^20^). Briefly, worms were treated with RNAi against a single gene throughout development, and adults were scored at multiple time points during adulthood for normal reporter protein levels, mitochondrial structure, and sarcomere structure. RNAi treatments that produced lethality or abnormal protein levels or structure were then applied to previously untreated, normal, adults to determine if the knockdown produced a defect solely due to a requirement of the gene during development or if the gene was also required for continued maintenance of fully developed muscle. Additionally, a key feature of the protein degradation screen was that RNAi treatments were not only identified as inducing altered proteostasis and increased protein degradation but they were also examined for the requirement of UNC‐51/ATG1 in producing the increased protein degradation and, if UNC‐51 was required, if increased autophagic vesicles were observed. This autophagy screen is graphically displayed in *Figure* 1B.

**Figure 2:**
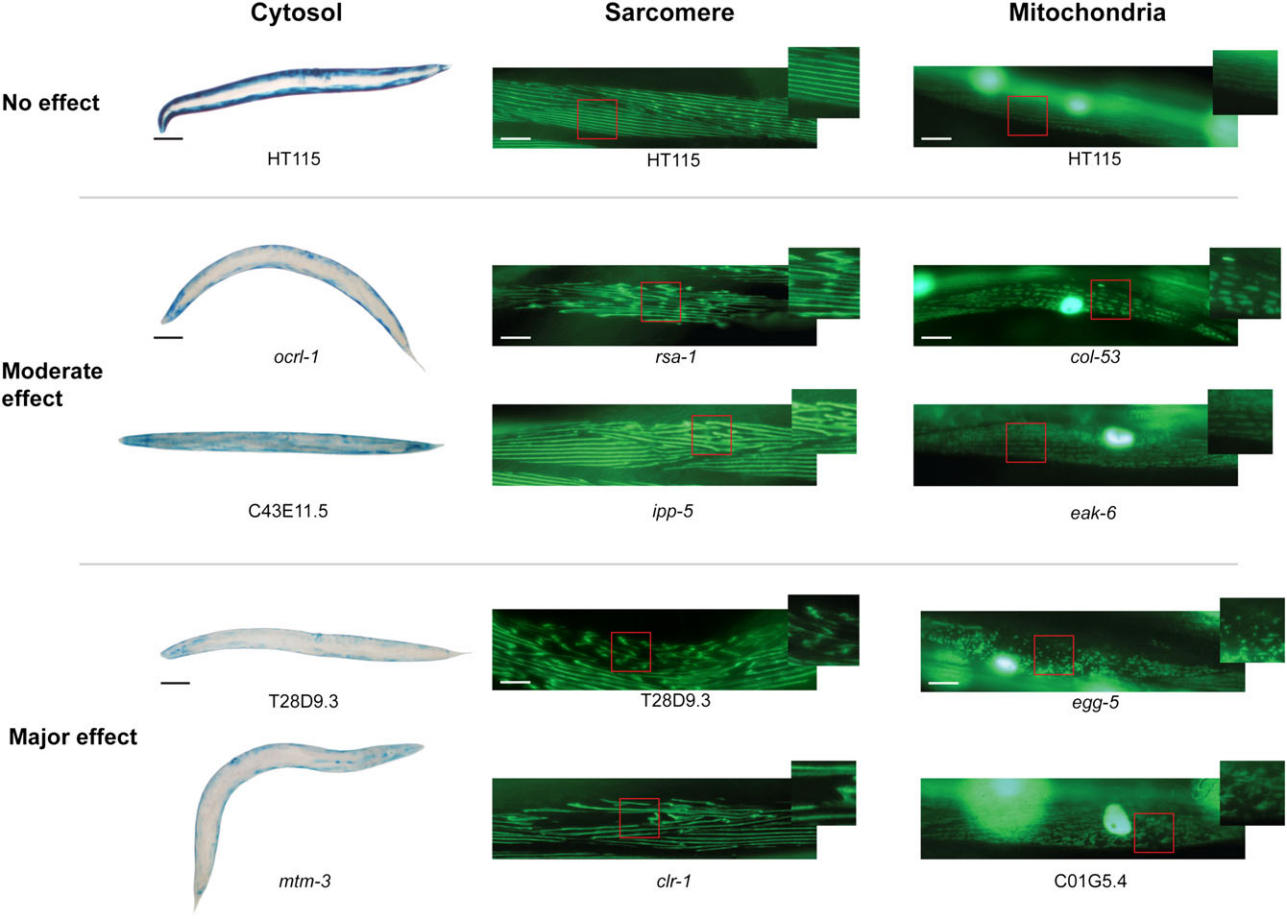
Examples of raw data from the screens for phosphatases required for normal muscle development and/or homeostasis. Images of sample phenotypes for proteostasis (cytosol), sarcomeres (sarcomere), and mitochondrial morphology (mitochondria). Empty vector control images are shown at the top with moderate and major defects shown below. Gene for which RNA interference produced the effect is noted below the image. The black scale bars represent 100 μm. The white scale bars represent 20 μm.

**Figure 3:**
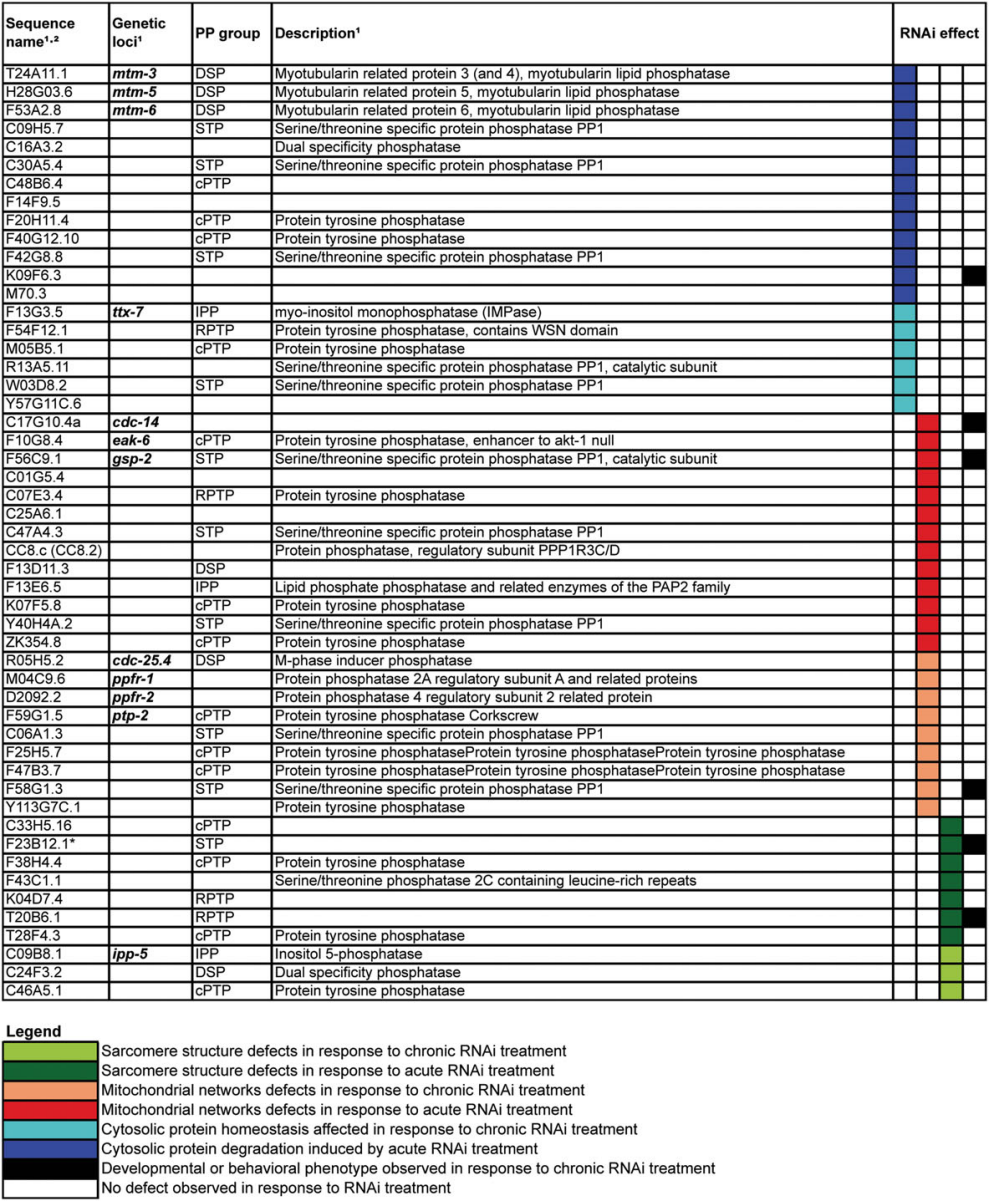
Phosphatases required for one aspect of normal muscle development and/or homeostasis. The same RNA interference screening protocol as used for the kinome requirement of a muscle was utilized^20^ with phosphatase‐encoding genes being targeted. Briefly, for chronic RNA interference treatment, four L4 larvae animals and two following generations of progeny were cultured on RNA interference bacteria clones. For both generations at 72–96 h after L4 transfer, progeny were observed on two consecutive days using microscopy for sarcomere structure, mitochondrial structure, or protein homeostasis. For acute RNAi treatment, synchronized adult worms grown on OP50 were transferred to RNAi bacteria seeded plates and observed at 24 h for structure and at 48 h and 72 h for all phenotypes. The impact of knockdown of phosphatases where a defect was noted in muscle is colour coded and displayed according to the inset legend, instances in which a whole animal defect was noted are indicated in black. Only RNA interference treatments that produced a defect in either protein homeostasis, mitochondrial structure, or sarcomere structure alone are displayed.

**Figure 4:**
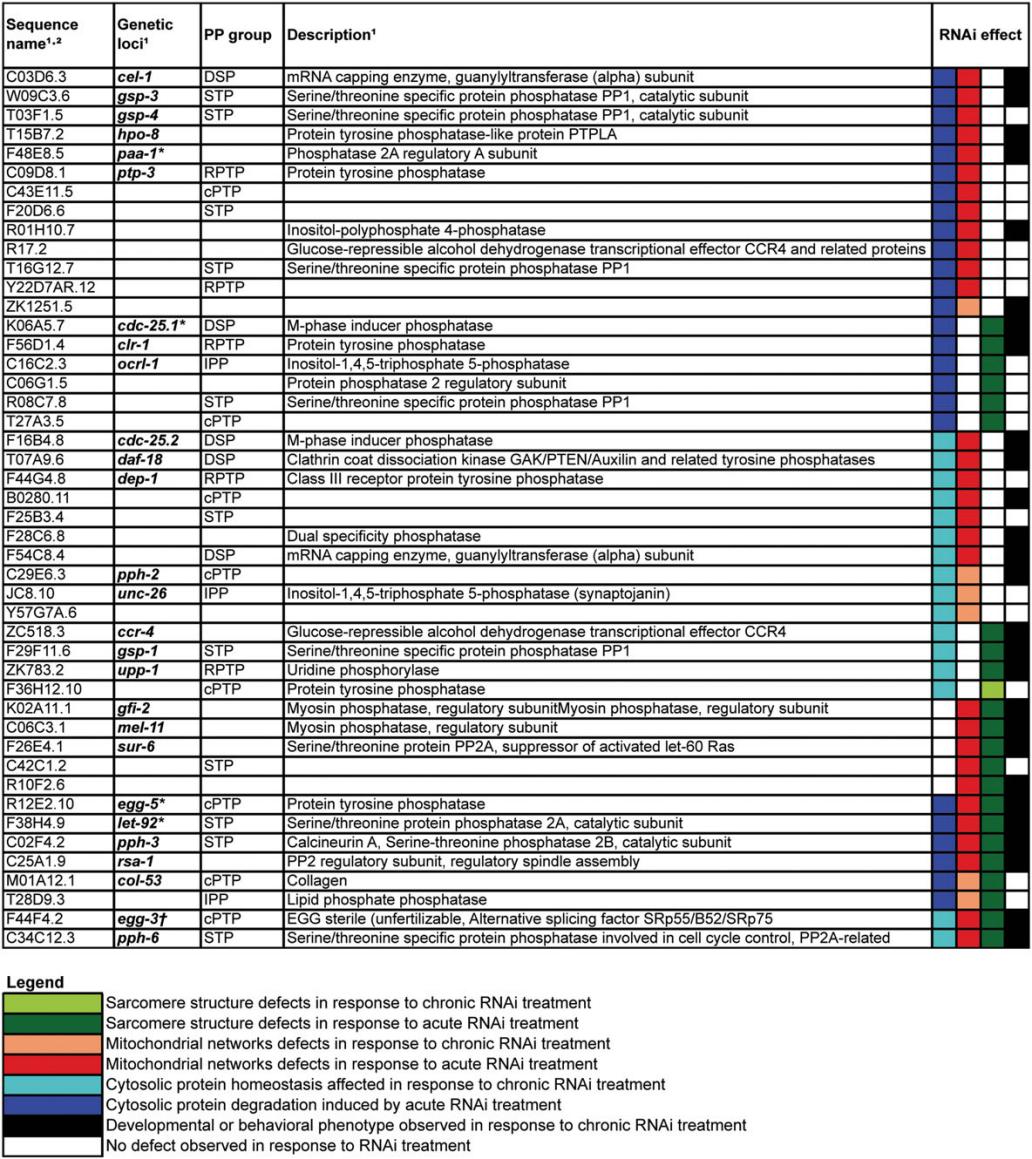
Phosphatases required for multiple aspects of normal muscle development and/or homeostasis. The same RNA interference screening protocol as used for the kinome requirement of muscle was utilized^20^ with phosphatase‐encoding genes being targeted. Briefly, for chronic RNA interference treatment, four L4 larvae animals and two following generations of progeny were cultured on RNA interference bacteria clones. For both generations at 72–96 h after L4 transfer, progeny were observed on two consecutive days using microscopy for sarcomere structure, mitochondrial structure, or protein homeostasis. For acute RNA interference treatment, synchronized adult worms grown on OP50 were transferred to RNA interference bacteria seeded plates and observed at 24 h for structure and at 48 and 72 h for all phenotypes. The impact of knockdown of phosphatases where a defect was noted in muscle is colour‐coded and displayed according to the inset legend, instances in which a whole animal defect was noted are indicated in black. Only RNA interference treatments that produced a defect in at least two of the subcellular phenotypes assayed (e.g. protein homeostasis, mitochondrial morphology, and sarcomere structure) are displayed. Genes for which chronic RNA interference induced an embryonic lethal phenotype in all three screens are labelled with asterisk; dagger indicates embryonic lethality only in the proteostasis screen.

As shown in *Figures* 2, 3, and 4, RNAi against 97 of 183 putative phosphatases produced a subcellular defect in a muscle. This suggests that roughly half of all phosphatases are required for normal development and/or maintenance of muscle. This percentage requirement is slightly higher than the roughly 40% of kinases that are required for normal development or maintenance of muscle and likely reflects the fact that because there are fewer phosphatases than kinases, there is less redundancy. Again, like the kinase requirement for muscle, more phosphatases are required for normal proteostasis than for mitochondrial structural homeostasis, and the least phosphatases are required for normal sarcomere homeostasis. This suggests that there are more signals impinging upon muscle metabolism than upon muscle sarcomere structure. Similarly to the kinase requirement for muscle, most phosphatases identified as required for normal development of muscle are also required for maintenance of adult muscle.

Included in the results are the identification of genes that were already known to regulate a muscle, such as a negative regulator of fibroblast growth factor receptor (FGFR), *clr‐1*,^14^, ^31^ and myosin phosphatase, *mel‐11*, which is known to be involved in elongation during development.^32^ These screens also identified embryonic lethality as expected for *let‐92* and *cdc‐25.1*. Although the identification of these genes appears to validate the RNAi results, not many of the other genes identified have been studied in detail or are known to regulate any of the processes examined. This was confirmed by gene ontology analysis using the online software DAVID,^33^ which failed to recognize a third of the genes we identified as having previously been assigned a biological function. This suggests that the approach taken in this study may be an important first step forward understanding the functions of previously unstudied phosphatase‐encoding genes. Interestingly, a little over half of the genes identified in these screens have homologues expressed in human skeletal muscle (see Supporting Information Data S1), suggesting that these genes may be candidates for further study of the regulation of muscle protein degradation, mitochondrial fission, and sarcomere maintenance in humans.

### Epistasis testing of potential degradation‐regulating phosphatases versus known signals

**Figure 5:**
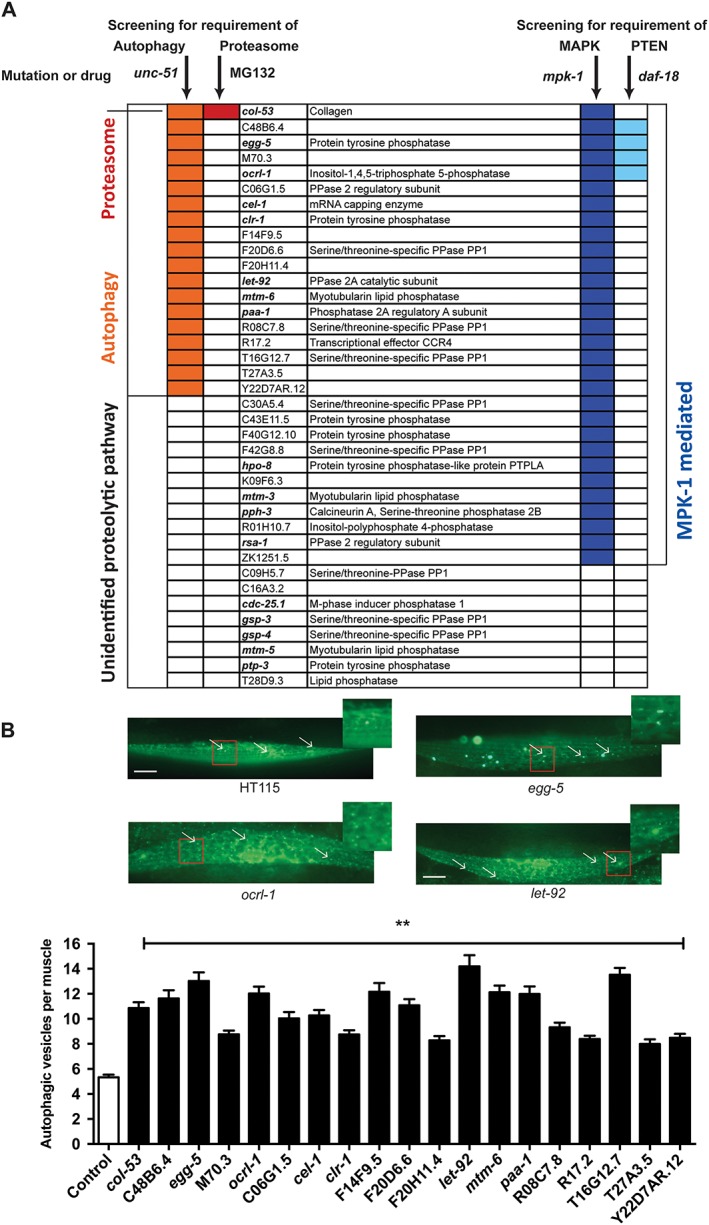
Autophagy is the most commonly triggered type of protein degradation in response to knockdown of a phosphatase. (A) Phosphatase‐encoding genes for which knockdown produced protein degradation were clustered into known proteolytic pathways and signalling mechanisms utilizing the same protocol as for the kinome requirement of a muscle.^20^ Briefly, knockdowns were examined for suppression of degradation in an autophagy mutant (*unc‐51*), in wild‐type animals treated with proteasome inhibitor (MG132), in a fibroblast‐growth factor pathway mutant (*mpk‐1*), and in an insulin‐growth factor pathway mutant (*daf‐18*). Colored boxes represent suppression of degradation in the mutant or treatment indicated at the top of the column. (B) Autophagic vesicles in muscle were assessed in untreated or phosphatase RNA interference‐treated animals as previously described for the kinome.^20^ Briefly, GFP::LGG‐1 containing worms we treated with empty vector or indicated phosphatase RNA interference and vesicles were counted. Top: sample images of empty vector control (top left) or RNA interference‐treated animal (top right and bottom left and right); white scale bars represent 20 μm. Bottom: quantification of three independent experiments (*n* = 20 each). Error bars indicate standard error of measurement. **P < 0.0001, one way ANOVA (graph pad prism).

To further identify how the RNAi knockdowns were producing cytosolic protein degradation, we functionally clustered the genes identified as required to prevent induction of protein degradation into those appearing to be required to prevent autophagy or proteasome‐mediated degradation. This was accomplished by treating *unc‐51* (ATG1) mutants or proteasome inhibitor‐treated animals with each RNAi treatment that induced protein degradation. Additionally, we used *mpk‐1* and *daf‐18* loss of function mutations to cluster these genes into FGFR‐mediated and IGFR‐mediated pathways, respectively.^13^ Half of the phosphatase‐encoding genes appear to be potential regulators of autophagy‐mediated protein degradation (*Figure* 5A), which is similar to the finding when the kinase‐encoding genes were previously knocked down. To confirm that autophagy was indeed induced in response to these RNAi treatments, we examined if GFP::LGG‐1 autophagic vesicles increased in muscle in response to treatment, which they did (*Figure* 5B). These findings suggest that when protein phosphorylation is perturbed either by increasing phosphorylation, in phosphatase RNAi knockdowns, or decreasing phosphorylation, in kinase knockdowns, that autophagy is triggered. In other words, autophagy appears to be sensitive to the global balance of numerous signals in muscle. Interestingly, most of the kinases and phosphatases that were identified to potentially regulate protein degradation required MPK‐1 (mammalian extracellular signal‐regulated kinase (ERK)). This suggests that MPK‐1 and other MAPKs may play a central role in the regulation of overall protein degradation within a cell. Given that ERK is known to be expressed and active in human skeletal muscle,^34^ perhaps a similar metabolic role for ERK in human skeletal muscle exists.

### Identification of *let‐92* as a putative central node for protein degradation

**Figure 6:**
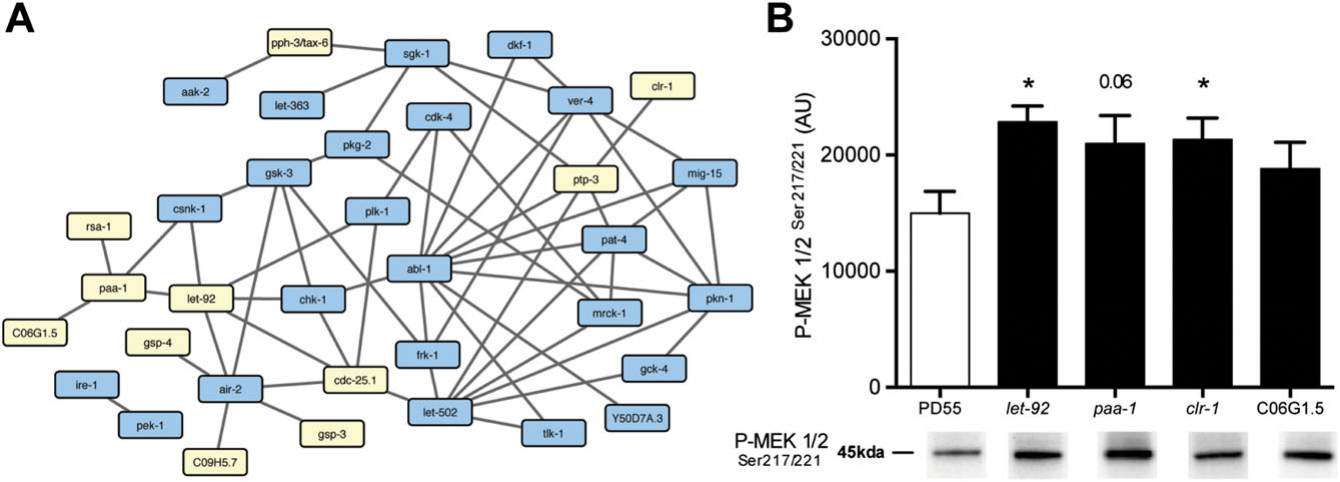
Functional interaction network of protein kinases and phosphatases required for normal protein degradation in muscle suggest that protein phosphatase 2A is a central node. (A) Kinases and phosphatases that were identified as required for lack of pathological protein degradation in muscle were examined for functional interactions in WormBase,^22^ GeneMANIA,^26^ and PhosphoPOINT.^27^ Kinases are indicated in blue and phosphatases in yellow. (B) Western blot analysis of MEK activation in response to knockdown of phosphatases identified in network analysis and as triggering autophagy. Quantification of MEK phosphorylation from three separate RNA interference experiments is displayed above representative blots. *P < 0.05, *t*‐test (graph pad prism).

To examine if the identified phosphatases and recently identified kinases that may regulate subcellular processes within muscle might act within a network regulating muscle homeostasis, we used past C. elegans genome‐wide known and predicted gene product physical interaction maps from published meta‐analyses,^35^, ^36^, ^37^ as well human kinome‐wide known gene product physical interaction data from a published meta‐analysis,^27^ to construct potential physical networks for the kinases identified in each screen. We also used past C. elegans genome‐wide known and predicted gene product functional interactions from published meta‐analyses,^35^, ^36^, ^37^ as well human kinome‐wide known gene product functional interaction data from a published meta‐analysis, to construct potential functional networks for the kinases identified in each screen. The physical networks are based upon binding data (e.g. yeast two hybrid, co‐immunoprecipitation) for the C. elegans kinase and/or data for the yeast, fly, rodent, and/or human orthologue^35^, ^36^, ^37^ while the functional networks are based upon limited genetic interactions for the C. elegans kinase and/or data for the yeast, fly, rodent, and/or human orthologue^35^, ^36^, ^37^ and a large amount of biochemical data for shared interacting phospho‐proteins for the human orthologue.^27^ Visualization of these predicted interactions using cytoscape did indeed reveal some potential interaction networks (see Supporting Information [Link], [Link]). Of note, there were not many known or predicted interactions between the phosphatases identified here. However, the combination of data on identified kinases and phosphatases resulted in a more integrated network than kinase or phosphatase‐specific networks alone. Also, within these potential networks emerged a phosphatase, *let‐92*, and kinase, *abl‐1*, that appeared to be central nodes as indicated by the number of connections to other identified genes (*Figure* 6A). The identification of such central nodes suggests one strategy in prioritizing phosphatases and kinases for further study.

### Knockdown of protein phosphatase 2A catalytic or regulatory subunit‐encoding genes results in increased MEK phosphorylation

Because LET‐92 appeared to be a central node and because PP2A is known to interact with Akt,^38^ a kinase known to control mammalian muscle size via both well‐appreciated^39^ and recently demonstrated mechanisms,^40^ we decided to further investigate the role of LET‐92 as a regulator of muscle protein degradation. The data presented in *Figure* 5 suggest that *let‐92* knockdown induces MAPK‐dependent autophagy. This is consistent with early reports of protein phosphatase 2A (PP2A) being a negative regulator of MAPK both *in vitro* 41 and in cultured cells^42^ and is also consistent with past reports of constitutive, autocrine, FGFR activation of Ras‐MAPK in C. elegans muscle being subject to negative regulation.^13^ Therefore, we tested if knockdown of PP2A catalytic and regulatory subunits resulted in increased phosphorylation of MEK, which should increase activation of MAPK. Western blots (*Figure* 6B) confirmed increased phosphorylation of MEK in response to knockdown of *let‐92*, *paa‐1*, and C06G1.5 as well as the *clr‐1* positive control. These results, coupled with those shown in *Figure* 5, suggest that PP2A is required to prevent excessive activation of autophagy in C. elegans muscle by modulating the activity of Ras‐MAPK signalling, which appears to act upstream of UNC‐51/ATG1.^13^

## Discussion

### Functional analysis of the phosphatome of Caenorhabditis elegans

Post‐translational modifications are a widely appreciated mechanism of modulating protein function. Phosphorylation is arguably one of the best studied such modifications, and the ability to modulate phosphorylation status of key proteins is clinically desirable.^43^, ^44^, ^45^ Much progress has been made on understanding the role that protein kinases play in phosphorylating their targets and in understanding the specificity of compounds against the kinome.^46^, ^47^ In contrast, the progress on understanding the role the protein phosphatases play in dephosphorylating their targets has lagged behind. Here, we have conducted three near full genome RNAi screen to identify phosphatases that when knocked down result in abnormal development and/or maintenance of muscle. Using this approach, we have found that roughly half of the phosphatome is required for normal development or maintenance of muscle. These data provide the first potential functional importance of more than a third of the C. elegans phosphatome and a preliminary picture of how many phosphatases are important for the proper development and maintenance of muscle. Further work is needed to determine if these phosphatases are required within muscle or other tissues for normal muscle health and to understand why and how these phosphatases are important. Given that putative human homologues of roughly half of the identified phosphatases are already known to be expressed in muscle (Supporting Information Data S1), it is likely that a good portion of the identified phosphatases act within muscle to modulate development and/or maintenance. While it is perhaps surprising that so many phosphatases appear to be required for normal development and/or maintenance of a muscle, the requirement is roughly similar to the kinome requirement for a muscle.^20^ The combined C. elegans phosphatome and kinome requirement for muscle provides a platform for future mechanistic studies of individual phosphatases and kinases, further unravelling of the complexity of the regulation of muscle, and a starting point for further therapeutic modulation of human muscle health.

### Disruption of phosphorylation events frequently triggers autophagy

Here, we have found that autophagic protein degradation is triggered in roughly half of individual phosphatase knockdowns that induce degradation. This result is intriguing for two reasons. First, as there are four major proteolytic systems in a muscle,^10^ this implies that a phosphatase is more likely to be important to prevent autophagy than to prevent proteasome‐meditated, caspase‐meditated, or calpain‐meditated degradation. Second, as knockdown of individual kinase‐encoding genes most frequently triggered autophagy,^20^ this implies that both increased and decreased phosphorylation events are likely to trigger autophagy. This finding from the combined work on the kinome and phosphatome suggests that autophagy is controlled by a balance of positive and negative signals and is consistent with past suggestions that in C. elegans, muscle autophagy is controlled by counterbalanced, constitutive pro‐degradation signalling from FGFR, and anti‐degradation signalling from insulin‐like growth factor receptor (IGFR).^13^ While the current observation is consistent with the past findings, what is surprising is the large extent to which both individual kinases and phosphatases appear to be required to prevent autophagy. One possible explanation for the more extensive requirement for kinases and phosphatases to prevent autophagy is that autophagy might be a default state that is subject to negative regulation in the presence of multiple signals that indicate favourable growth conditions. Such a notion is consistent with the previous suggestion that mTor is an integrator of multiple favourable growth conditions to modulate both protein synthesis and degradation.^48^, ^49^ This also raises the question of the relative importance of autophagic‐mediated as opposed to proteasome‐mediated protein degradation for maintaining human muscle homeostasis.

### Mitogen‐activated protein kinase as a central regulator of protein degradation

In addition to finding that autophagic protein degradation is the type of protein degradation most commonly triggered in response to knockdown of any individual kinase or phosphatase, we have found that functional MPK‐1 is very frequently required for the protein degradation that is triggered in response to knockdown of any individual kinase^20^ or phosphatase (*Figures* 3 and 4). Thus, analysis of both the kinome and phosphatome suggests a central role of MPK‐1 in modulating muscle protein degradation in response to phosphorylation events. This observation, like the observation of both increased and decreased phosphorylation events being associated with increased autophagy, suggests that perhaps a central integrator of multiple favourable growth conditions exists. Our connectivity analysis of the kinome and phosphatome with respect to protein degradation suggests that LET‐92 is a central node and that it appears to be a modulator of muscle protein degradation with knockdown producing *mpk‐1*‐dependent autophagic degradation. These results, coupled with the fact that ERK is known to be expressed and active in human skeletal muscle,^34^ raise the question of if Raf‐MAPK is a central modulator of autophagic degradation, with a significant number of kinases and phosphatases providing modulatory signals for this central pathway. This also raises the question of if Raf‐MAPK is not just a central player in controlling protein synthesis but also of autophagy, perhaps acting to either modulate or complement a similar role of mTor. Thus, our results from C. elegans open the door to further mechanistic studies of the regulation of human muscle metabolism.

### Potential implications for human health and disease

We have identified phosphatases that are required for normal muscle health in a worm. Eighty of these phosphatases have human counterparts and 53 are already known to be expressed in human muscle. If they control human muscle health like they do worm muscle health, then these phosphatases are important for normal muscle health and may contribute to human muscle disease; translational work that remains to be completed. This has several implications for the clinic. First, these phosphatases, like the previously uncovered kinases,^20^ are potential druggable targets for therapeutic intervention in muscle health. For example, as has recently been reported for mouse muscle, stimulation of protein kinase A results in increased proteasome‐mediated protein degradation, whereas treatment with protein phosphatase 1 decreases proteasome‐mediated protein degradation.^50^ Thus, with further work, it is highly probable that protein kinase and phosphatase inhibitors can be used to modulate protein degradation levels in either direction, work that will no doubt be accelerated by the cancer field's push to identify effective protein kinase and phosphatase inhibitors that are safe for human use.^51^, ^52^ Inhibition/activation of kinases and phosphatases may also prove useful in other respects. For example, the phosphatase PTPH1 is known to regulate p97,^53^ which has recently been suggested to extract proteins from the highly organized, protein dense sarcomeres.^54^ Therefore, clinical modulation of multiple molecular processes within human muscle is likely to be achievable just by targeting these two classes of druggable proteins. Second, drugs that are used to target protein phosphatases or kinases in other diseases, for example cancer, may produce myopathy as a side effect due to the normal role of the phosphatase or kinase in muscle health. For example, inhibition of the protein kinase MEK produces rhabdomyolysis^55^ and is known to be important for worm muscle health.^14^ Third, mutations in protein phosphatases or kinases may account for some rare as yet molecularly uncharacterized muscular dystrophies. For example, mutations in the phosphatase myotubularin 1 are known to cause X‐linked myotubular myopathy^56^ and a mutation in the phosphatase myotubularin‐releated protein 14 has been shown to cause centronuclear myopathy.^57^ Fourth, declines in expression of phosphatases or kinases with age may contribute to the onset and/or progression of sarcopenia. For example, myotubularin‐releated protein 14 displays reduced expression with age in mice and its loss accelerates sarcopenia.^58^ Lastly, alterations in expression of phosphatases or kinases with activity may contribute to individual differences in muscular adaptation to exercise. For example, the kinase MARCKS and phosphatase PTEN display increased expression following a programme of resistance exercise training.^59^ Given that inactivity is one of the top non‐communicable diseases in the world,^60^ this suggests a substantive new avenue of research into combating the negative muscular consequences of inactivity, the impact of phosphatase or kinase modulators on muscular adaptation to activity.

## Conflict of interest

Susann Lehmann, Joseph J Bass, Thomas F Barratt, Mohammed Z Ali, and Nathaniel J Szewczyk declare that they have no conflict of interest.

## Supporting information


**Dataset 1.** Full list of phosphatases screened.

**Dataset 2.** List of kinase kinase interactions.

**Dataset 3.** List of phosphatase phosphatase interactions.

**Dataset 4.** List of combined kinase phosphatase interactions.
